# Structural and Electrochemical Characterization of Zn_1−x_Fe_x_O—Effect of Aliovalent Doping on the Li^+^ Storage Mechanism

**DOI:** 10.3390/ma11010049

**Published:** 2017-12-29

**Authors:** Gabriele Giuli, Tobias Eisenmann, Dominic Bresser, Angela Trapananti, Jakob Asenbauer, Franziska Mueller, Stefano Passerini

**Affiliations:** 1 School of Science and Technology-Geology Division, University of Camerino, Via gentile III da Varano, 62032 Camerino, Italy; 2 Helmholtz Institute Ulm (HIU), Helmholtzstrasse 11, 89081 Ulm, Germany; tobias.eisenmann@kit.edu (T.E.); jakob.asenbauer@kit.edu (J.A.); franziska-mueller1@gmx.de (F.M.); stefano.passerini@kit.edu (S.P.); 3 Karlsruhe Institute of Technology (KIT), P.O. Box 3640, 76021 Karlsruhe, Germany; 4 School of Science and Technology-Physics Division, University of Camerino, Via Madonna delle Carceri, 62032 Camerino, Italy; angela.trapananti@unicam.it

**Keywords:** lithium-ion battery, anode, crystal chemistry, electrochemistry

## Abstract

In order to further improve the energy and power density of state-of-the-art lithium-ion batteries (LIBs), new cell chemistries and, therefore, new active materials with alternative storage mechanisms are needed. Herein, we report on the structural and electrochemical characterization of Fe-doped ZnO samples with varying dopant concentrations, potentially serving as anode for LIBs (Rechargeable lithium-ion batteries). The wurtzite structure of the Zn_1−x_Fe_x_O samples (with x ranging from 0 to 0.12) has been refined via the Rietveld method. Cell parameters change only slightly with the Fe content, whereas the crystallinity is strongly affected, presumably due to the presence of defects induced by the Fe^3+^ substitution for Zn^2+^. XANES (X-ray absorption near edge structure) data recorded ex situ for Zn_0.9_Fe_0.1_O electrodes at different states of charge indicated that Fe, dominantly trivalent in the pristine anode, partially reduces to Fe^2+^ upon discharge. This finding was supported by a detailed galvanostatic and potentiodynamic investigation of Zn_1−x_Fe_x_O-based electrodes, confirming such an initial reduction of Fe^3+^ to Fe^2+^ at potentials higher than 1.2 V (vs. Li^+^/Li) upon the initial lithiation, i.e., discharge. Both structural and electrochemical data strongly suggest the presence of cationic vacancies at the tetrahedral sites, induced by the presence of Fe^3+^ (i.e., one cationic vacancy for every two Fe^3+^ present in the sample), allowing for the initial Li^+^ insertion into the ZnO lattice prior to the subsequent conversion and alloying reaction.

## 1. Introduction

Rechargeable lithium-ion batteries (LIBs) are now the technology of choice for electrochemical energy storage, and are consequently employed in a wide range of devices ranging from portable electronics to electric vehicles and stationary energy storage [1,2,3,4]. However, in order to eventually achieve the highly challenging goal of fully replacing gasoline-powered vehicles by electric ones, both the energy density and power density require further improvement [5]. With respect to the anode side, the state-of-the-art active material graphite has essentially reached its limits in terms of specific capacity and charge rate capability [6,7]. Therefore, new cell chemistries and, accordingly, new anode materials, presumably based on alternative lithium-ion storage mechanisms, are being presently investigated. The two most prominent ones in this context are conversion- and alloying-type materials [6,8,9,10].

Both mechanisms, however, suffer from substantial intrinsic drawbacks, such as large volume changes upon (de-)lithiation in the case of alloying materials (leading to short cycle life) [8,10] or high voltage hysteresis in the case of conversion materials [9]. As an example, pure ZnO, a material widely investigated for semiconductors [11], suffers from essentially irreversible Li_2_O formation upon initial lithiation when applied as an anode material in LIBs. Hence, it typically offers low specific capacities of around 330 mAh g^−1^, solely originating from the reversible Li-Zn de-/alloying reaction (theoretical specific capacity of 988 mAh g^−1^, if fully reversible) [12]. When Zn is partially substituted by transition metals (e.g., Fe, Co) within the wurtzite structure, however, the reversibility of the Li_2_O formation is substantially enhanced [12,13,14]. Consequently, transition metal-doped zinc oxides combine both conversion and alloying mechanisms in one active material and are accordingly classified as conversion-alloying materials (CAMs) [14]. Several studies have already revealed the outstanding electrochemical performance of, for instance, carbon-coated Fe-doped ZnO, when applied as negative electrode materials in LIBs, delivering reversible specific capacities of almost 1000 mAh g^−1^ [12,15]. Nevertheless, the exact role of the transition metal for the lithiation mechanism in CAMs, enabling the reversible formation and decomposition of Li_2_O, has still not been fully unraveled.

Herein, we report the detailed investigation of the structural and electrochemical properties of Zn_1−x_Fe_x_O (with x = 0, 0.02, 0.04, 0.06, 0.08, 0.10, and 0.12) by means of X-ray diffraction (XRD), X-ray absorption spectroscopy (XAS), galvanostatic cycling, and cyclic voltammetry. The results suggest that an increasing amount of cationic vacancies as a result of the substitution of Zn^2+^ by Fe^3+^ leads to a substantial lithium ion insertion into the wurtzite lattice, accompanied by the reduction of Fe^3+^ to Fe^2+^, prior to the subsequent—in such case facilitated—conversion/alloying reaction.

## 2. Results and Discussion

### 2.1. Synthesis and Basic Characterization

**Figure A1:**
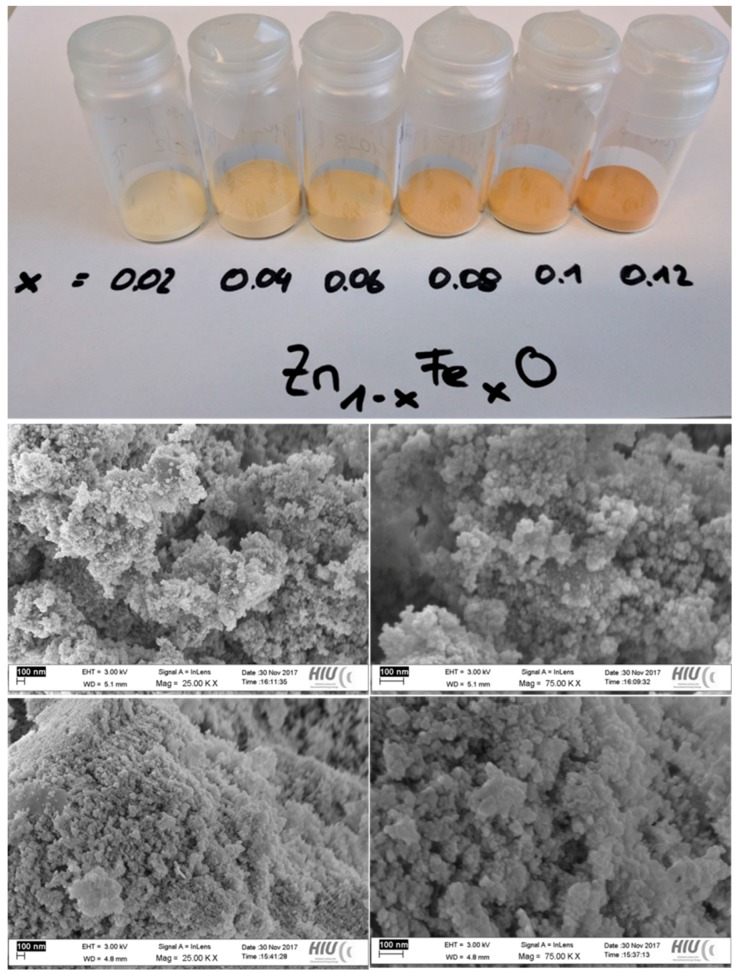
**Top panel**: Photograph of the Zn_1−x_Fe_x_O samples with x being 0.02, 0.04, 0.06, 0.08, 0.10, and 0.12. The photograph nicely illustrates the increasing orange coloring with an increasing Fe content. **Lower panels**: Scanning electron microscopy (SEM) micrographs of selected Zn_1−x_Fe_x_O samples with x being 0.12 (**middle left and right**) and 0.04 (**bottom left and right**).

The synthesis of the Zn_1−x_Fe_x_O sample series was conducted following a synthesis route developed earlier [12]. In excellent agreement with a previous study [15], the color of the samples varied, becoming increasingly orange for increasing iron concentration. The particle size was well in the range of a few nm (10–20 nm—depending on the Fe content; see Appendix A Figure A1). The Zn:Fe ratio was determined by means of inductively coupled plasma optical emission spectroscopy (ICP-OES), confirming the targeted Fe concentrations of x = 0.02, 0.04, 0.06, 0.08, 0.10, and 0.12 within an experimental error of around 1%.

### 2.2. Powder XRD Characterization and Structural Refinement

**Figure 1:**
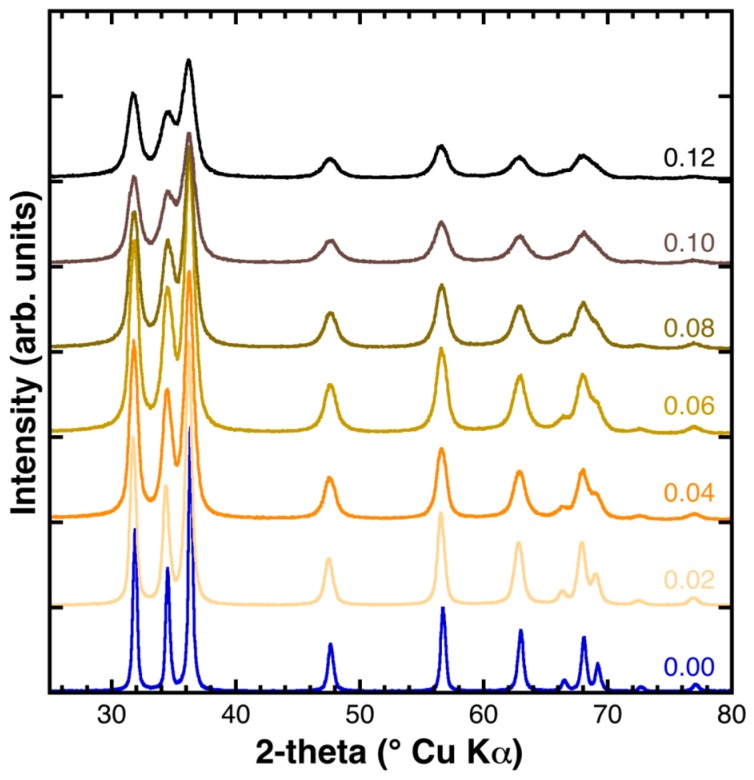
Powder X-ray diffraction (XRD) patterns of the as synthesized samples. The Fe fraction is indicated at the right side above each diffractogram.

The X-ray diffraction patterns of the as synthesized samples are shown in Figure 1, arranged from bottom to top for increasing Fe content. All the diffraction reflections could be indexed according to the hexagonal ZnO structure (*P6*_3_*mc* space group), whereas the absence of other reflections allowed the exclusion of the presence of crystalline impurities such as other zinc and/or iron oxide phases. The increase of the diffraction reflections’ Full Width at Half Maximum (FWHM) was evident (from bottom to top), revealing that the ZnO crystallinity and/or crystallite size was remarkably affected by the Fe concentration.

**Figure 2:**
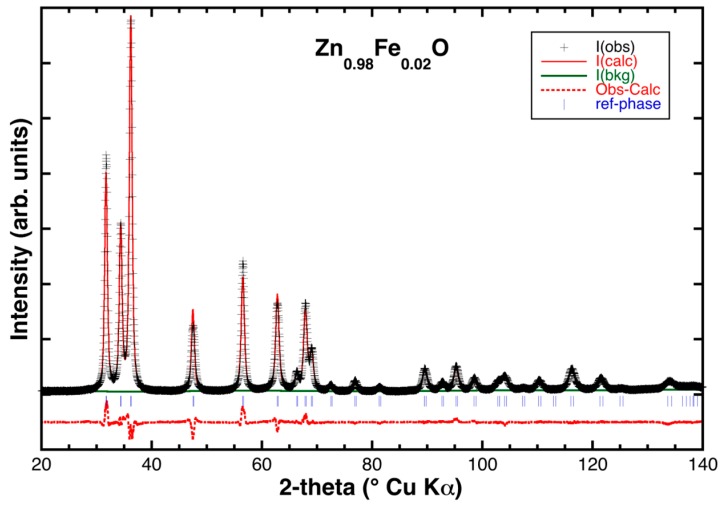
Typical Rietveld refinement of a Fe-doped sample (Zn_0.98_Fe_0.02_O): Black crosses mark the experimental data; the solid red and green lines represent the theoretical pattern and background function, respectively, while the dotted red line is the residual. Vertical blue lines mark the angular positions of the ZnO reflections.

Rietveld refinement provided an accurate determination of the unit cell parameters and atomic positions for all the samples. Table 1 summarizes the disagreement indexes, the most relevant structural parameters, and the average crystallites sizes (calculated using the Williamson-Hall (W.-H.) method [16]), whereas in Figure 2, a typical comparison between observed and calculated XRD patterns is displayed.

**Figure 3:**
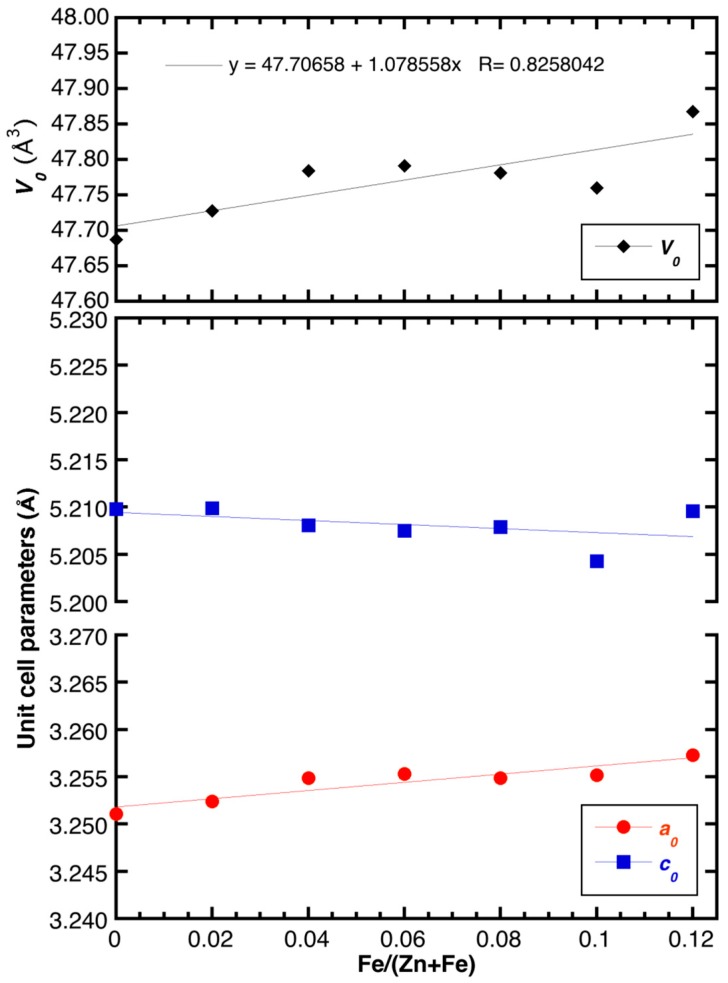
Refined unit cell parameters of the as synthesized samples (error bars shown within symbols) as a function of the Fe/(Fe + Zn) ratio. The linear fits for the refined data are also shown.

**Figure A2:**
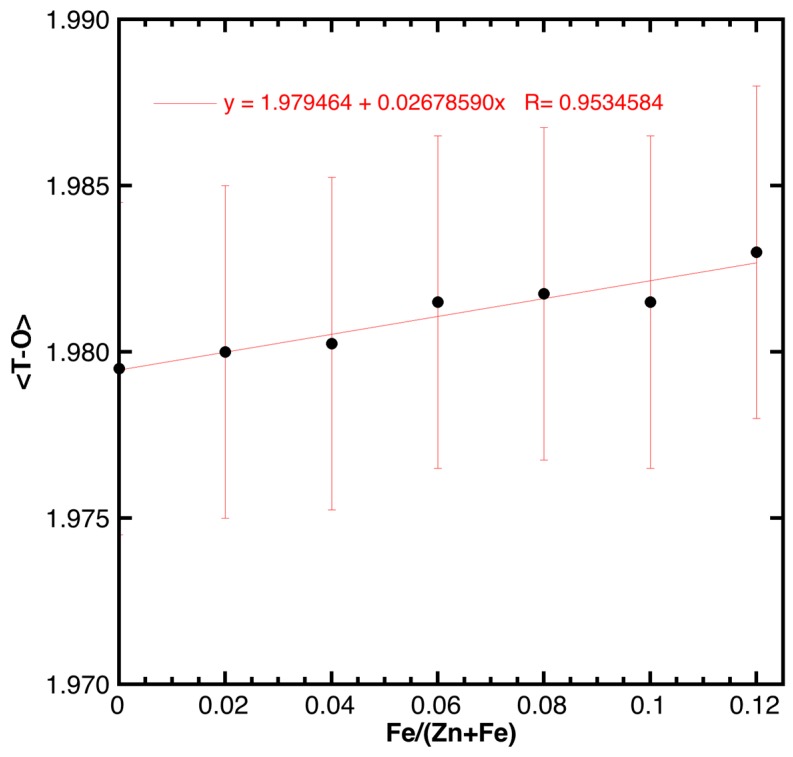
<T–O> distance as a function of the Fe concentration.

The absence of any relevant amounts (i.e., at the 0.5 wt % level) of additional Fe-bearing phases in the XRD patterns of the doped samples suggests that Fe was successfully inserted as dopant into the ZnO lattice in the whole Fe concentration range analyzed here (x ranging between 0 and 0.12). Similar to previously published data [17], the substitution of Zn by Fe resulted in a small but appreciable variation of the unit cell volume (Figure 3), while the variation of the <T–O> distances was within the experimental error (Figure A2 in the Appendix A). In this regard, it is interesting to notice that the unit cell volume of the doped samples increased—albeit only slightly—as a function of the Fe content despite the fact that Fe^3+^ in tetrahedral coordination had a smaller ionic radius than Zn^2+^ (i.e., 0.49 and 0.60 Å, respectively [18]). In particular, the increase of the *a*_0_ parameter could be fitted by a linear trend *a*_0_ = 3.2518(6) + 0.043(9)X_Fe_ (R = 91.4%), for which the slope of the linear function is about four times its standard deviation and thus, significant. Contrarily, the *c*_0_ parameter could also be fitted by a linear trend *c*_0_ = 5.2094(13) − 0.021(18)X_Fe_ (R = 47.1%), but the slope of this linear function had a value comparable to its standard deviation and was therefore not significant. In sum, the unit cell volume displayed a slight increase with the iron content according to the following function: *V*_0_ = 47.706(24) + 1.08(33)X_Fe_ (R = 82.6%). As a conclusion, we assigned this anisotropic variation of the unit cell volume to two driving forces: the Fe doping and the presence of structural defects as a result of the aliovalent substitution of Zn^2+^ by Fe^3+^ [17].

**Figure 4:**
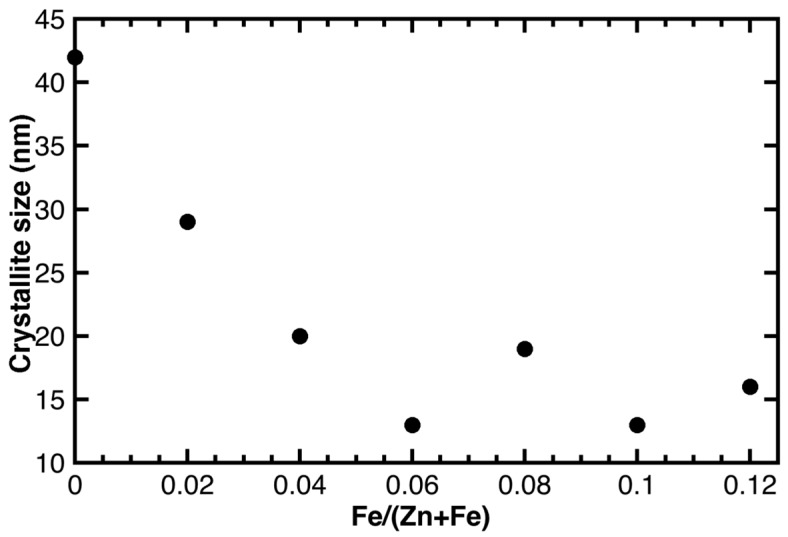
Crystallite size vs. Fe content of the as synthesized samples, revealing a marked decrease in crystallite size specifically for Fe concentrations as small as 0.02 and 0.04, whereas for higher Fe contents the decrease in crystallite size is less pronounced.

The FWHM of the XRD reflections increases progressively with the increase of the Fe content, x from 0 to 0.12, meaning that the crystallinity of the samples decreases in the same order. The reflection shape parameters obtained from the Rietveld refinement were used to determine the FWHM as a function of 2-theta and to build the corresponding W.-H. plots. The crystallite size, calculated by means of the Scherrer formula based on the intercepts of the W.-H. plots, decreased from 42 to 13 nm (Figure 4), whereas the strain displayed a more scattered trend. In absence of a careful calibration of the instrumental parameters of the utilized diffractometer, these values have to be taken solely as indicative and should not be considered as an absolute measure of the crystallite size. However, we noted that these values were of the same order of magnitude as those obtained earlier by means of transmission electron microscopy (TEM) and small-angle X-ray scattering (SAXS) for the same sample series [15]. 

These results obtained for the series of zinc oxide samples with varying Fe dopant concentrations, ranging from 0 to 0.12, indicated that the doping remarkably affected the crystallinity. This effect was particularly pronounced for Fe contents as small as x = 0.02 and 0.04, while it was less pronounced for higher Fe contents. A similar effect of the Fe content on the crystallite size was also observed by Kumar et al. for Fe-doped ZnO samples (with x ranging from 0 to 0.06), prepared by a sol-gel synthesis method [19], and Reddy et al. for example (with x ranging from 0 to 0.05) synthesized by a low-temperature combustion route [20].

It is worth mentioning once more that the cell parameter variations observed here were not solely related to the ionic radius of the substituting cation (0.49 vs. 0.60 for tetrahedrally coordinated Fe^3+^ vs. Zn^2+^, respectively [18]). In fact, besides displaying compositional variations, the samples studied here also showed amply varying degrees of crystallinity and, possibly, presence of defects such as cationic vacancies and/or interstitial oxygen [17]. As already reported in the literature for other oxide systems [21], both crystallinity and defects content could strongly contribute to alter unit cell parameters, but not in a predictable way.

### 2.3. Fe K-Edge XANES Spectroscopy

**Figure 5:**
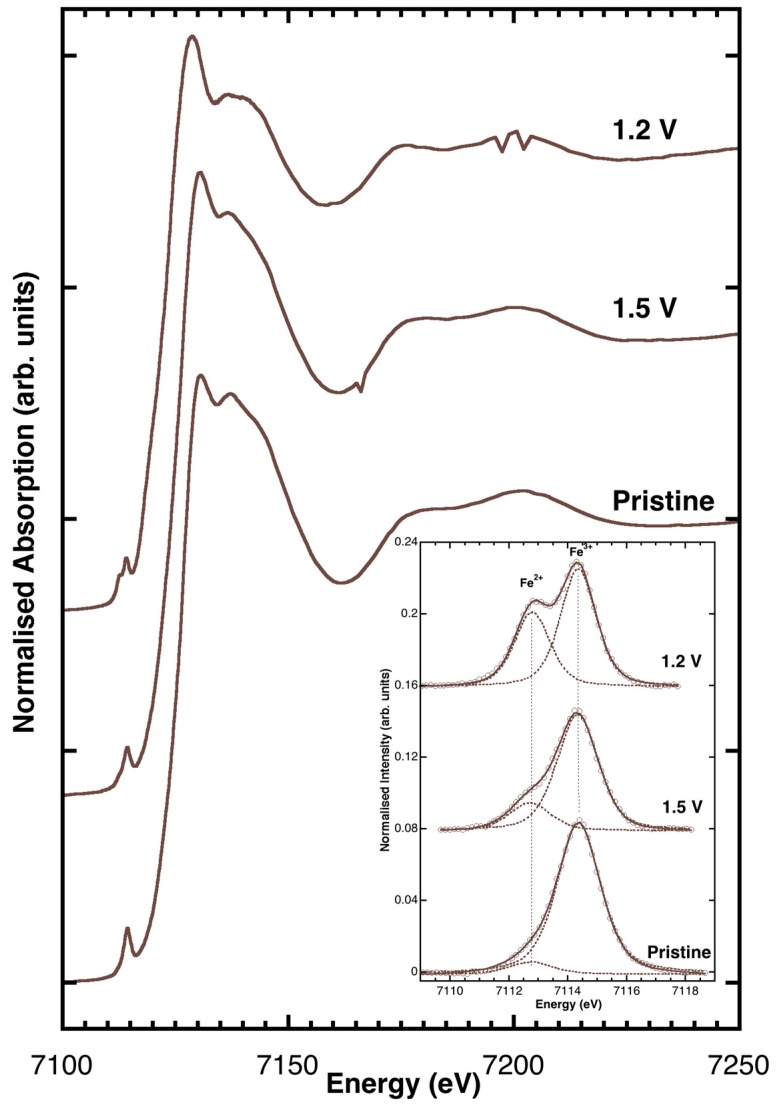
Fe K-edge XANES (X-ray absorption near edge structure) spectra measured ex situ for carbon-coated Zn_0.9_Fe_0.1_O anodes before electrochemical testing (pristine) and discharged to 1.5 and 1.2 V vs. Li^+^/Li.

X-ray absorption near edge structure (XANES) data collected at the Fe K-edges are presented in Figure 5. The XANES spectra were measured ex situ for various anode samples of carbon-coated Zn_0.9_Fe_0.1_O. The pristine sample, i.e., a non-cycled electrode, was comparable to that reported in Giuli et al. [17], whereas those ones discharged to 1.5 and 1.2 V vs. Li^+^/Li displayed a slight shift of the edge energy toward lower energy. The background subtracted pre-edge peaks (shown in the inset of Figure 5) also had similar shapes and intensities. However, while the pre-edge peak of pristine Zn_0.9_Fe_0.1_O displayed a major component at 7114.4 eV (typical of Fe^3+^), the spectrum of Zn_0.9_Fe_0.1_O/C discharged to 1.5 and 1.2 V had a clear component at ca. 7112.7 eV, revealing the presence of a significant fraction of Fe^2+^. 

Comparing the pre-edge peak integrated intensities and energy centroids with those of model compounds from literature [16,22,23,24] provides Fe^3+^/(Fe^2+^ + Fe^3+^) ratios = 0.95(5) for the pristine Zn_0.9_Fe_0.1_O/C., 0.75(7) and 0.5(7) for the samples discharged at 1.5 and 1.2 V, respectively.

### 2.4. Electrochemical Characterization

**Figure 6:**
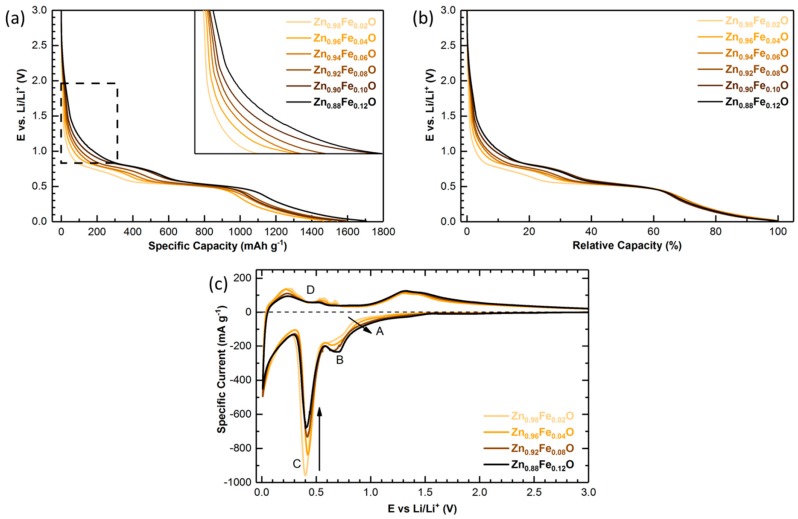
(**a**) Potential profiles for the first galvanostatic discharge of Zn_1−x_Fe_x_O-based electrodes (x = 0.02, 0.04, 0.06, 0.08, 0.10, and 0.12), applying a discharge rate of 0.05C (i.e., 48.3 mA g^−1^); the inset shows a magnification of the area highlighted by the dashed box; (**b**) The same potential profiles as presented in (**a**), but plotted versus the normalized capacity; (**c**) Cyclic voltammetry conducted for selected samples (x = 0.02, 0.04, 0.08, and 0.12), applying a sweeping rate of 50 µV s^−1^.

In order to complement these structural results with the electrochemical lithium storage behavior of Zn_1−x_Fe_x_O as a function of the iron content, galvanostatic cycling was conducted. The results for the initial lithiation are depicted in Figure 6a. All samples essentially revealed the typical potential profile of Fe-doped ZnO, characterized by two distinct potential plateaus at ~0.8 V and ~0.5 V and a subsequent smooth potential decrease to 0.01 V, selected as the cathodic cut-off potential [12]. As determined earlier by means of in situ XRD, the lithiation mechanism includes the conversion of Zn_0.9_Fe_0.1_O to Zn^0^, Fe^0^, and Li_2_O as well as the alloying of Zn and Li [12].

**Figure 7:**
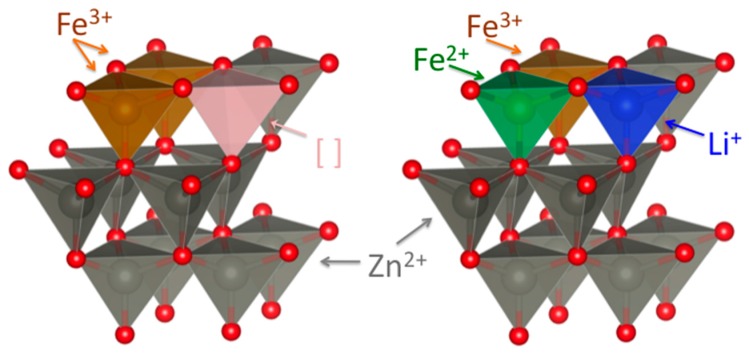
Schematic illustration of the cationic vacancies in the wurtzite lattice of the pristine material due to the Fe^3+^ for Zn^2+^ substitution (**left panel**) and the resulting initial lithium ion insertion in the vacant tetrahedral sites at higher potentials, leading to the partial reduction of Fe^3+^ to Fe^2+^ (**right panel**).

Interestingly, a significantly different behavior was observed for the high-potential region during the initial lithiation, i.e., the potential region from the open circuit voltage down to 0.8 V—or in terms of specific capacity for the first 100–350 mAh g^−1^ (indicated by the dashed box and magnified in the inset of Figure 6a). The Li-storage capacity in this region increased with increasing iron content. Combining the previously reported in situ XRD data [12], the herein presented XRD results (indicating an increasing amount of structural defects as a result of the substitution of Zn^2+^ by Fe^3+^), and the XANES results (revealing the reduction of Fe^3+^ to Fe^2+^ at potentials of 1.5 and 1.2 V), the increasing capacity contribution observed at high potentials could be ascribed to Li^+^ insertion into the wurtzite lattice favored by the presence of cationic vacancies and accompanied by the reduction of Fe^3+^ to Fe^2+^, as schematically illustrated in Figure 7.

Interestingly, also the shape of the first potential plateau changed as a function of the Fe content (Figure 6a,b). While the Zn_1−x_Fe_x_O samples with x from 0.04 to 0.12 showed a comparably well-defined plateau, though shifted to lower potentials for decreasing x, the sample with x = 0.02 revealed a rather sloped shape. Also, the overall capacity obtained at the end of this first potential plateau decreased with a decreasing iron content, providing about 425, 475, 550, 575, 630, and 660 mAh g^−1^ for x = 0.02, 0.04, 0.06, 0.08, 0.10, and 0.12, respectively. Considering that iron is completely reduced to the metallic state at this potential (as revealed by the in/ex situ XANES analysis, to be published soon) and that the overall contribution of the conversion reaction to the theoretical capacity is 666 mAh g^−1^, these findings indicated that the conversion reaction was kinetically favored in case of high Fe concentrations, presumably as a result of the relatively larger amount of initially inserted lithium. For lower iron contents, the conversion reaction was accordingly completed along the second potential plateau, i.e., together with the occurring alloying reaction [12]. This is confirmed by the plot of the normalized capacity in Figure 6b, revealing the same capacity values for all samples at the end of this second plateau. As a matter of fact, such a mixed potential for the reduction of the transition metal dopant and zinc was observed also for Co-doped ZnO [13], which did not show any significant cationic vacancies, allowing for an initial Li^+^ insertion, due to the divalent oxidation state of cobalt [17].

The general capacity excess upon discharge compared to the theoretical maximum for this reaction (ca. 966 mAh g^−1^) was largely assigned to the relatively low first cycle coulombic efficiency of 61–64%, indicating a significant electrolyte decomposition, especially at lower potentials.

The general trend of a relatively increasing capacity at higher potentials (regions A and B in Figure 6c) and relatively decreasing capacity at lower potentials (region C in Figure 6c) for increasing x was very well observed when comparing the cyclic voltammograms (CVs) in Figure 6c. While we may refer to a previous publication [15] for the detailed discussion of the CVs, it appears noteworthy that Zn_0.98_Fe_0.02_O reveals the typical de-alloying peaks (region D), commonly observed for pure ZnO [13,15], suggesting that the upon lithiation formed zinc nanograins are comparably larger for such a rather low iron content [13].

In conclusion, both galvanostatic cycling and cyclic voltammetry highly suggested that the defects in the wurtzite structure were cationic vacancies, allowing for the initial Li^+^ insertion into these vacancies. This lithium insertion resulted in increasing capacities at higher potentials for increasing Fe content, accompanied by the reduction of Fe^3+^ to Fe^2+^, and followed by the kinetically favored conversion reaction. 

## 3. Materials and Methods

### 3.1. Material Synthesis

Zn_1−x_Fe_x_O (with x = 0.02, 0.04, 0.06, 0.08, 0.10, 0.12) was synthesized according to a previously reported method [15]. Zinc (II) gluconate (Alfa Aesar, Lancashire, UK) and iron (II) gluconate (Sigma Aldrich, St. Louis, MO, USA) were dissolved in ultra-pure water with respect to the desired dopant ratio (0.2 M total ion concentration). This solution was added dropwise to a second solution comprising 1.2 M sucrose, and the obtained solution was stirred for 15 min. Subsequently, the solvent was evaporated at 160 °C and the obtained material was further heated to 300 °C in order to decompose the sucrose and dry the precursor. Finally, the solid powder was grinded and calcined in a tubular furnace (Nabertherm, L9/12/P330, Lilienthal, Germany) at 450 °C for 3 h (3 K min^−1^ heating rate). ICP-OES was conducted in order to determine the metal ion concentrations by dissolving the samples in hot hydrochloric acid and via double determination in a Spectro Arcos from Spectro Analytical Instruments (Kleve, Germany) with axial plasma view. Scanning electron microscopy (SEM) was performed by means of a Zeiss LEO 1550 (Zeiss, Oberkochen, Germany).

### 3.2. Powder XRD Characterization and Structural Refinement

The crystal structure of the as-synthesized samples was characterized by powder XRD with an automated Philips Bragg-Brentano diffractometer equipped with a graphite monochromator. The long-fine focus Cu tube was operated at 40 kV and 25 mA. Spectra were recorded in the 2θ range 20–140° with a 0.03° step and 14 s counting time. The structures were refined with the program General Structure Analysis System (GSAS) [25]. The reflection shape was modeled with a Pseudo-Voigt function; the FWHM was refined as a function of 2θ taking into account both Gaussian and Lorentzian broadening. The refinement was carried out for the space group *P6*_3_*mc* and the starting atomic coordinates were those of Xu and Ching [26] with the initial values for isotropic temperature factors (*Uiso*), arbitrarily chosen as 0.025 Å^2^. The O atom sites were designated as fully occupied, while constrains for fractional occupancies for Fe and Zn were used according to the stoichiometry of the synthesized samples. The background was modeled with a 9-terms polynomial function. Cell parameters, scale factor, and the background polynomial function were free variables during the refinement. Parameters were added stepwise to the refinement in the following order: 2θ zero-shift, peak shape, peak asymmetry, atomic coordinates, and isotropic thermal factor. The intensity cut-off for the calculation of the profile step intensity was initially set at 1.0% of the peaks maxima and were lowered to 0.1% in the final stages of the refinements. Final convergence was assumed to be reached when the parameter shifts were <1% of their respectively estimated standard deviation. Estimated errors, provided by the Rietveld refinement program, are ±0.0002 Å for the cell parameters and ±0.002 Å for the selected interatomic distances. However, the error calculation is probably over-optimistic, as it does not include the correlation among parameters and other error sources (like the overlapping of many diffraction reflections, for instance). In order to get an alternative estimate of the accuracy of the refined structural data, we have compared the set of structural parameters obtained using different refinement strategies for the same diffraction data. These comparisons show that realistic estimates of the error bars are ±0.0005 Å for the cell parameters and ±0.005 Å for the selected interatomic distances. Trials of refinements were also done assuming some of the iron could be located in interstitial sites. However, the resulting disagreement indexes were higher than in the case of Fe location in the Zn site. Thus, we assume that all the iron is substituting for Zn and that no significant amount of iron is located in interstitial sites [17].

### 3.3. XAS Data Collection and Analysis

Ex situ Fe K-edge XAS spectra were measured on cycled electrodes (section below) at beamline BM08 [27] of the European Synchrotron Radiation Facility (ESRF, Grenoble, France). A fixed-exit double-crystal Si(311) monochromator, operated in flat crystals mode, was used to select the energy of the beam delivered by the bending magnet source. Higher order harmonics were rejected using two Pd-coated mirrors working at an incidence angle of 3.6 mrad. The second mirror was left unbent. The beam size at the sample was about 2 mm × 2 mm FWHM. XAS spectra were measured in transmission mode using ionization chambers filled with N_2_ and Ar gases at pressures tuned to achieve the optimal efficiency at the Fe K-edge absorption edge energy (20% and 80% of absorption of the incident beam respectively). The monochromator energy was calibrated by setting the first inflection point of the edge of metal Fe to 7112 eV. The spectrum of a metal Fe foil placed downstream of the main experimental chamber was collected simultaneously with any anode spectrum to monitor and correct the energy scale against possible monochromator instabilities. Fe K-edge pre-edge peak analysis was carried out following a standard procedure reported elsewhere [22,23]. The pre-edge peak was fitted by a sum of pseudo-Voigt functions and their intensities along with energy positions were compared with those of Fe model compounds from literature in order to extract the information on the absorber oxidation state in the cycled samples. More details on the pre-edge peak fitting method can be found in references [24,28].

### 3.4. Electrochemical Characterization

Electrodes were prepared with a dry composition of 75 wt % active material, 20 wt % Super C65 (Imerys, Paris, France) and 5 wt % sodium carboxymethyl cellulose (CMC; Dow-Wolff Cellulosics, Bollitz, Germany) dissolved in ultra-pure water. Slurries were homogenized in a planetary ball mill (Fritsch Vario-Planetary Mill Pulverisette 4, Fritsch GmbH, Idar-Oberstein, Germany) for 3 h using 12 mL zirconia jars and zirconia balls. Subsequently, the slurries were cast onto dendritic copper foil (Schlenk, Bitterfeld, Germany) at 50 mm s^−1^ with a doctor blade (BYK Additive & Instruments, Wesel, Germany) and a wet film thickness of 120 µm. The obtained electrode batches were dried at room temperature overnight before punching 12 mm disc electrodes and drying them in a vacuum glass oven (Büchi B585, Büchi, Rungis, France) at 120 °C for 24 h. All electrochemical experiments were conducted in three-electrode Swagelok-type cells. The prepared electrodes were used as working electrodes, while lithium foil (Honjo Metal Co., Higashi-Osaka, Japan) served as counter and reference electrode. Glass fiber sheets (Whatman GF/D, Whatman, Maidstone, UK), drenched in a 1 M solution of LiPF_6_ in ethylene carbonate and diethyl carbonate (3:7 by wt), were used as separators. Galvanostatic cycling was conducted on a Maccor Series 4300 battery cycler, applying cut-off potentials of 3.0 V and 0.01 V vs. Li^+^/Li. All applied currents refer to a theoretical specific capacity of 966 mAh g^−1^ (1C = 966 mA g^−1^). For cyclic voltammetry experiments, a galvanostatic-potentiostatic VMP multichannel cycler (Biologic Science Instruments, Seyssinet-Pariset, France) was used, applying a sweeping rate of 50 µV s^−1^ and reversing potentials of 3.0 V and 0.01 V vs. Li^+^/Li.

For the ex situ XAS measurements, electrodes based on carbon-coated Zn_0.9_Fe_0.1_O active material were prepared on carbon paper, serving as current collector, and, apart from the pristine sample, cycled in three-electrode Swagelok-type cells according to the same procedures as above. Subsequently, the electrodes were recovered at discrete stages (1.5 V and 1.2 V), washed with dimethyl carbonate to remove the electrolyte, and sealed within polyethylene (PE) foil in an Ar-filled glove box to avoid air contamination.

## 4. Conclusions

Fe-doped Zn_1−x_Fe_x_O samples (with x ranging from 0.00 to 0.12) have been successfully synthesized using a self-developed synthesis route. Powder XRD reveals all samples to be phase-pure, having the same wurtzite structure of pristine ZnO. Galvanostatic cycling and cyclic voltammetry data of Fe-doped ZnO electrodes highlight an increasing capacity contribution in the initial stages of lithiation, which is ascribed to the reduction of trivalent to divalent Fe, as also supported by the ex situ XANES data. In sum, the data reported herein indicate that increasing iron dopant contents lead to increasing cationic vacancies in the lattice, allowing for the electrochemical insertion of Li^+^ into wurtzite-structured ZnO, thus, kinetically favoring the subsequent conversion reaction. We may, hence, anticipate that these results will further enlighten the role of non-divalent transition metal dopants in ZnO and, in general, aliovalent transition metal dopants for conversion/alloying materials (CAMs) in general, ideally paving the way for the development of new CAMs with further enhanced energy and power densities.

## Figures and Tables

**Table 1:** Structural parameters, average crystallite size, and disagreement indexes of the Rietveld refinements performed using isotropic temperature factors and isotropic broadening of the diffraction reflections. The errors of the Unit cell parameters and <T–O> distances are provided in brackets.

	ZnO	Zn_0.98_Fe_0.02_O	Zn_0.96_Fe_0.04_O	Zn_0.94_Fe_0.06_O	Zn_0.92_Fe_0.08_O	Zn_0.9_Fe_0.1_O	Zn_0.88_Fe_0.12_O
*a*_0_ (Å)	3.2511 (3)	3.2524 (1)	3.2549 (1)	3.2553 (1)	3.2549 (2)	3.2552 (2)	3.2573 (2)
*c*_0_ (Å)	5.2098 (1)	5.2099 (1)	5.2081 (3)	5.2075 (3)	5.2079 (5)	5.2043 (4)	5.2096 (4)
*V*_0_ (Å^3^)	47.687 (1)	47.728 (1)	47.784 (3)	47.791 (3)	47.781 (5)	47.760 (5)	47.868 (4)
<T-O>	1.9795 (50)	1.9800 (50)	1.9802 (50)	1.9815 (50)	1.9818 (50)	1.9815 (50)	1.9830 (50)
wRp	8.36	7.11	7.44	7.88	7.88	7.60	8.7.44
Rp	6.60	5.64	5.79	6.26	6.07	5.87	5.92
R_F_^2^	3.49	3.43	4.51	5.09	3.78	4.19	4.26
R_F_	1.92	1.79	2.59	3.05	2.02	2.30	2.17
*a*_0_/*c*_0_	0.6240	0.6188	0.6250	0.6251	0.6250	0.6255	0.6252
W.-H. intercept ^1^	0.0033	0.0048	0.0069	0.0104	0.0063	0.011	0.0088
W.-H. slope ^1^	0.0003	0.0013	0.0023	0.0006	0.0045	0.0031	0.0046
Crystallite size (nm)	42	29	20	13	22	13	16

^1^ Fitted intercepts and slope of the W.-H. plots, obtained using the refined peak shape parameters.

## References

[1] Tarascon J.-M., Armand M. (2001). Issues and challenges facing rechargeable lithium batteries. Nature.

[2] Armand M., Tarascon J.-M. (2008). Building better batteries. Nature.

[3] Scrosati B., Garche J. (2010). Lithium batteries: Status, prospects and future. J. Power Sources.

[4] Dunn B., Kamath H., Tarascon J.-M. (2011). Electrical Energy Storage for the Grid: A Battery of Choices. Science.

[5] Thackeray M.M., Wolverton C., Isaacs E.D. (2012). Electrical energy storage for transportation—Approaching the limits of, and going beyond, lithium-ion batteries. Energy Environ. Sci..

[6] Aravindan V., Lee Y.S., Madhavi S. (2015). Research Progress on Negative Electrodes for Practical Li-Ion Batteries: Beyond Carbonaceous Anodes. Adv. Energy Mater..

[7] Yamada Y., Iriyama Y., Abe T., Ogumi Z. (2009). Kinetics of Lithium Ion Transfer at the Interface between Graphite and Liquid Electrolytes: Effects of Solvent and Surface Film. Langmuir.

[8] Zhang W.J. (2011). A review of the electrochemical performance of alloy anodes for lithium-ion batteries. J. Power Sources.

[9] Cabana J., Monconduit L., Larcher D., Palacín M.R. (2010). Beyond Intercalation-Based Li-Ion Batteries: The State of the Art and Challenges of Electrode Materials Reacting Through Conversion Reactions. Adv. Mater..

[10] Obrovac M.N., Chevrier V.L. (2014). Alloy Negative Electrodes for Li-Ion Batteries. Chem. Rev..

[11] Özgür Ü., Alivov Y.I., Liu C., Teke A., Reshchikov M.A., Doǧan S., Avrutin V., Cho S.J., Morko̧ H. (2005). A comprehensive review of ZnO materials and devices. J. Appl. Phys..

[12] Bresser D., Mueller F., Fiedler M., Krueger S., Kloepsch R., Baither D., Winter M., Paillard E., Passerini S. (2013). Transition-Metal-Doped Zinc Oxide Nanoparticles as a new Lithium-Ion Anode Material. Chem. Mater..

[13] Mueller F., Geiger D., Kaiser U., Passerini S., Bresser D. (2016). Elucidating the Impact of Cobalt Doping on the Lithium Storage Mechanism in Conversion/Alloying-Type Zinc Oxide Anodes. ChemElectroChem.

[14] Bresser D., Passerini S., Scrosati B. (2016). Leveraging valuable synergies by combining alloying and conversion for lithium-ion anodes. Energy Environ. Sci..

[15] Mueller F., Gutsche A., Nirschl H., Geiger D., Kaiser U., Bresser D., Passerini S. (2017). Iron-Doped ZnO for Lithium-Ion Anodes: Impact of the Dopant Ratio and Carbon Coating Content. J. Electrochem. Soc..

[16] Mote V., Purushotham Y., Dole B. (2012). Williamson-Hall analysis in estimation of lattice strain in nanometer-sized ZnO particles. J. Theor. Appl. Phys..

[17] Giuli G., Trapananti A., Mueller F., Bresser D., Dácapito F., Passerini S. (2015). Insights into the Effect of Iron and Cobalt Doping on the Structure of Nanosized ZnO. Inorg. Chem..

[18] Shannon R.D. (1976). Revised Effective Ionic Eadii and Systematic Studies of Interatomic Distances in Halides and Chalcogenides. Acta Crystallogr. Sect. A.

[19] Kumar S., Mukherjee S., Singh R.K., Chatterjee S., Ghosh A.K. (2011). Structural and optical properties of sol-gel derived nanocrystalline Fe-doped ZnO. J. Appl. Phys..

[20] Reddy A.J., Kokila M.K., Nagabhushana H., Sharma S.C., Rao J.L., Shivakumara C., Nagabhushana B.M., Chakradhar R.P.S. (2012). Structural, EPR, photo and thermoluminescence properties of ZnO:Fe nanoparticles. Mater. Chem. Phys..

[21] Hazen R.M., Jeanloz R. (1984). Wüstite (Fe_1−x_O): A review of its defect structure and physical properties. Rev. Geophys..

[22] Wilke M., Farges F., Petit P.E., Brown G.E., Martin F. (2001). Oxidation state and coordination of Fe in minerals: An Fe K-XANES spectroscopic study. Am. Mineral..

[23] Giuli G., Pratesi G., Cipriani C., Paris E. (2002). Iron local structure in tektites and impact glasses by extended X-ray absorption fine structure and high-resolution X-ray absorption near-edge structure spectroscopy. Geochim. Cosmochim. Acta.

[24] Giuli G., Pratesi G., Eeckhout S.G., Koeberl C., Paris E., Gibson R.L., Reimold W.U. (2010). Iron reduction in silicate glass produced during the 1945 nuclear test at the Trinity site (Alamogordo, New Mexico, USA). Large Meteorite Impacts and Planetary Evolution IV.

[25] Larson A.C., Von Dreele R.B. (2004). General Structure Analysis System (GSAS).

[26] Xu Y.-N., Ching W.Y. (1993). Electronic, optical, and structural properties of some wurtzite crystals. Phys. Rev. B.

[27] D‘Acapito F., Trapananti A., Torrengo S., Mobilio S. (2014). X-ray Absorption Spectroscopy: The Italian Beamline GILDA of the ESRF. Not. Neutroni Luce Sincrotrone.

[28] Giuli G., Cicconi M.R., Paris E. (2012). The ^[4]^Fe^3+^–O distance in synthetic kimzeyite garnet, Ca_3_Zr_2_ [Fe_2_SiO_12_]. Eur. J. Mineral..

